# Some OFF bipolar cell types make contact with both rods and cones in macaque and mouse retinas

**DOI:** 10.3389/fnana.2014.00105

**Published:** 2014-09-26

**Authors:** Yoshihiko Tsukamoto, Naoko Omi

**Affiliations:** ^1^Studio RetinaNishinomiya, Japan; ^2^Department of Biology, Hyogo College of MedicineNishinomiya, Japan

**Keywords:** primate, monkey retina, synapse, mesopic vision, gap junctions, serial section electron microscopy, parallel processing, retinal neural circuits

## Abstract

This study compared the types of OFF bipolar cells found in the macaque retina with those found in the mouse retina and determined whether these OFF bipolar cells make direct contacts with both rods and cones by serial section transmission electron microscopy. We performed scatter plots and cluster analysis of the morphological variables of their axon terminals such as the stratification level, the arbor thickness, the arbor area, and the number of ribbons. Five OFF bipolar cell types, including the recently discovered DB3b type, were identified in the macaque retina. The macaque OFF bipolar cell types FMB, DB1, DB2, DB3a, and DB3b corresponded to the mouse OFF bipolar cell types 2, 1, 4, 3a, and 3b, respectively. In addition to contacting rod bipolar cells, ~7% of rods in the macaque retina made basal contacts exclusively with one cell type, DB3b, whereas 18% of rods in the mouse retina made basal contacts with one or two of types, 3a, 3b, and 4. Approximately 3% of mouse rods were divergently connected to two OFF bipolar cells of different types, but macaque rods were solely connected to one OFF bipolar cell. Rod-rod gap junctions were localized at rod cell bodies and axons in the outer nuclear layer in both macaque and mouse retinas. The direct rod-OFF bipolar connection system is slightly more developed in the mouse retina than in the macaque retina, possibly as a fine-tuned adaptation to nocturnal conditions. This one-step direct synaptic pathway from rods to OFF bipolar cells may enhance the response speed to OFF light stimuli compared with more indirect pathways via rod-cone gap junctions (a two-step pathway) and via rod bipolar and AII amacrine cells (a three-step pathway).

## Introduction

A direct synaptic connection from the rod photoreceptor cells to certain types of OFF (cone) bipolar cells has been found in mice, rats, rabbits, and cats (Soucy et al., 1998; Hack et al., 1999; Tsukamoto et al., 2001; Fyk-Kolodziej et al., 2003; Li et al., 2004; Protti et al., 2005; Pang et al., 2010, 2012) as the third rod signaling pathway in the mammalian retina. The first pathway, via rod bipolar and AII amacrine cells, and the second pathway, via rod-cone gap junctions, are present in primates as well as in many other mammals (Raviola and Gilula, 1973; Kolb and Famiglietti, 1974; McGuire et al., 1984; Dacheux and Raviola, 1986; Strettoi et al., 1990; Grünert and Martin, 1991; Wässle et al., 1991, 1995; DeVries and Baylor, 1995), but it is unknown whether this third pathway exists in the primate retina.

Boycott and Wässle (1991) classified macaque OFF bipolar cells into one flat midget bipolar (FMB) type and three diffuse bipolar (DB) types DB1, DB2, and DB3. The DB3 cell type was subsequently further classified into types DB3a and DB3b by Puthussery et al. (2013), based on immunohistochemical and electrophysiological differences. DB3a and DB3b cells have axon terminals at nearly the same level of stratification, but the axonal arbor of DB3a cells is much larger than that of DB3b cells (Puthussery et al., 2013). Thus, the DB3a type appears to correspond to the DB3 type originally described, whereas the DB3b type is thought to be novel. Although Hopkins and Boycott (1997) reported many instances of synaptic contact of multiple bipolar cell types with cones, no instances with rods were found in the macaque retina. In this study, we detail the synaptic connection of DB3b cells to rods.

In mice, OFF bipolar cells that directly contact both rods and cones have been identified immunocytochemically as types 3a (T3a), 3b (T3b) (Mataruga et al., 2007), and 4 (T4) (Haverkamp et al., 2008). In the previous article by Tsukamoto et al. (2001), all mouse OFF bipolar cells that contact both rods and cones were collectively classified as type B2; all others contacting cones only were classified as type B1. Type B2 must therefore contain T3a, T3b, and T4 cells; type B1 must T1 and T2 cells, according to the current classification. In accordance with the most recent taxonomy of mouse bipolar cells (Euler et al., 2014), in this article we refer to the mouse OFF bipolar cell types as type 1 (T1), type 2 (T2), etc.

Tsukamoto et al. (2001) showed that rods that make contacts with OFF bipolar cells are coupled with several neighboring rods by gap junctions, possibly to integrate rod signals, in the mouse retina. Consistent with this finding, Hornstein et al. (2005) demonstrated tracer and signal coupling between rods in the macaque retina, where rod-rod gap junctions were found in regions containing rod somas and passing rod axons. Not necessarily contradictory to this finding, Raviola and Gilula (1973) found rare gap junctions between rod spherules in the macaque retina. Taken together, these two studies imply that rod-rod gap junctions are present in the outer nuclear layer (ONL) but absent from the outer plexiform layer (OPL) in the macaque retina. In the mouse retina, Tsukamoto et al. (2001) observed rod-rod gap junctions between soma and terminal (spherule), terminal and terminal, and axon and terminal via use of a series of tangential sections that contained the OPL and a lower part of the ONL. In summary the authors illustrated cone-cone, rod-cone, rod-rod gap junctions at different heights (in this order) from the OPL toward the ONL (Tsukamoto et al., 2001), but that study did not precisely describe the location of those gap junctions. Thus, the localization of rod-rod gap junctions remains to be determined.

In this study, we first identified and classified all types of OFF bipolar cells via scatter plots and clustering of 3D-reconstructed cells in the macaque and mouse retinas. This procedure enables us to identify homologous types in these species. Next, we examined gap junctions between cones and cones, cones and rods, and rods and rods in macaque and mouse retinas, but we particularly focused on the localization of rod-rod gap junctions between the OPL and ONL. Last, we examined how common rod-OFF bipolar synapses are in these species by determining the percentage of rods making contact with OFF bipolar cells. Comparing the frequency, distribution, and microcircuit organization of the rod-OFF bipolar pathways in macaque and mouse retinas may yield important clues for understanding the evolutionary background of the retinal circuitry in these species.

## Materials and methods

### Tissue preparation and electron microscopy

#### Macaque

The posterior retina of the right eye of a Japanese monkey (*Macaca fuscata*, 7 years old, female, 6.5 kg) was used for serial section transmission electron microscopy. The animal was preliminarily anesthetized with an intramuscular injection of 3.5 mL ketamine HCl solution (10 mg/mL), laid on a warm carpet, ventilated via a tracheotomy tube, and then deeply anesthetized with intravascular perfusion of sodium pentobarbital (10 mg/mL) with monitoring for blood pressure (80–120 mmHg). The vitreous body in front of the fovea of the right eye was replaced with saline using a vitrectomy apparatus (Omni-Surge 5000, Inami, Tokyo, Japan). The animal was first intravascularly perfused with a solution of 0.9% NaCl and 0.1% heparin in 0.1 M phosphate buffer (PB, pH 7.4) for 5 min, followed by perfusion of 1 L of 1% glutaraldehyde plus 1% paraformaldehyde in PB. At the same time, 5 mL of 3% glutaraldehyde plus 3% paraformaldehyde in PB were perfused into the eye using the same syringe as that used for the vitrectomy. Another 1.7 L of 3% glutaraldehyde plus 3% paraformaldehyde in PB was then perfused intravascularly. This procedure was performed in compliance with the Guide for the Care and Use of Experimental Animals (Hyogo College of Medicine).

After decapitation, the eye was enucleated and hemisectioned through the equatorial plane. After rinsing with PB, several areas of the retina along the horizontal meridian were cut together with the choroid and sclera to protect the retina from mechanical deformation. These tissue pieces were post-fixed with 2% osmium tetroxide and 1% potassium ferricyanide in PB for 2 h in a refrigerator, stained *en bloc* with 3% uranyl acetate in 80% methanol, dehydrated with ethanol, and embedded in araldite (Nisshin EM, Tokyo, Japan). A series of 817 radial sections 90 nm in thickness (73.5 μm in total thickness) was taken from the block containing the retina at 2.9–3.4 mm temporal to the foveal center. These sections were mounted on 120 formvar-coated single-slot grids and stained with 3% uranyl acetate in 80% methanol and Reynolds' lead citrate. Electron micrographs of the series were acquired first at 400× using the JEM 1220 electron microscope (Jeol Ltd., Tokyo, Japan) at the Joint-Use Research Facilities of Hyogo College of Medicine. A total of 24 overlapping images were acquired from each individual section at 3000×, which captured a rectangular area of 90 × 187 μm covering from the OPL to the ganglion cell layer (GCL) using a montage system of 4 × 6 negatives. These images were enlarged four-fold; thus, the final magnification of prints used for image analysis was 12000×. This series was used for the examination of OFF bipolar cells and junctions between rod spherules. When the sites of candidate gap junctions were identified, additional electron micrographs were taken at 40000× with various tilting angles to reveal the characteristic structures of these gap junctions.

#### Mice

A series of 366 radial sections were prepared from the central area of the posterior retina of a C57BL/6J, 9-week-old, female mouse (20 g; SLC, Shizuoka, Japan), which is the same series that was used previously (Tsukamoto et al., 2001). This series was used for the examination of OFF bipolar cells and junctions between adjacent rod spherules. Another series of 133 tangential sections including the ONL were prepared from the posterior retina of a C57BL/6J, 8-week-old, male mouse (25 g; SLC, Shizuoka, Japan) for examination of rod-rod gap junctions in the ONL. This is different from the previous series of tangential sections used by Tsukamoto et al. (2001). Procedures for electron microscopy were similar to those described for the macaque retina above; these procedures were described in more detail by Tsukamoto et al. (2001).

### Examination area of the macaque retina

The angular separation between the temporal edge of the optic disk and the foveal center is 15° in rhesus monkeys (*Macaca mulatta*), as measured by Weiskrantz and Cowey (1967) and de Monasterio et al. (1985). The corresponding distance in our sample eye was 3.18 mm. Using these data under the assumption that *M. fuscata* and *M. mulatta* have similar gross retinal structures, the conversion of retinal distance to visual angle is 212 μm/°. The examination area was located 3.00–3.25 mm temporal to the foveal center, and the center of this area was ~15° away from the foveal center.

**Figure 1:**
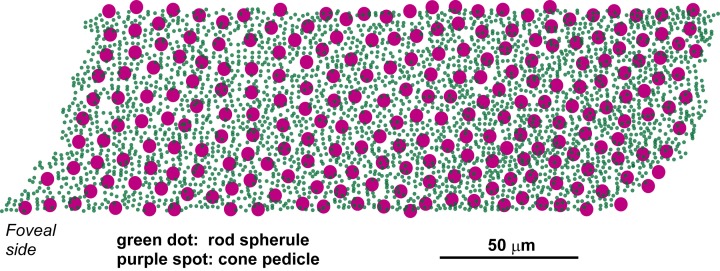
**Distribution of cone pedicles and rod spherules within the area of highest rod density in the macaque (*M. fuscata*) retina**. This parallelogram-shaped area is located 3.00–3.25 mm from, or approximately at a visual angle of 15° temporal to, the foveal center. The montage of high-magnification micrographs used for reconstruction covers the central rectangular area of 73.5 × 187 μm of this parallelogram.

The top-view distribution of 3159 rod spherules and 237 cone pedicles (Figure 1) was reconstructed from electron micrograph prints acquired at 4000× (10× enlargement of 400× negatives). This survey area, which formed a rough parallelogram of 73.5 × 224 μm with an irregular contour located 3.00–3.25 mm temporal to the foveal center, was measured to be 0.01684 mm^2^ (Image-J; NIH, USA). Half of the cells that extended across the edge of the parallelogram were subtracted from the total number for density measurements. Thus, the corrected total numbers of rods and cones in this region were 2889 and 212, respectively, and the corresponding densities were 172 × 10^3^ spherules/mm^2^ and 12.6 × 10^3^ pedicles/mm^2^, respectively. The density ratio of rods to cones was 13.6.

The retinal neural circuitry is thought to be constrained by the density ratios of constituent neurons which in turn depend on the retinal eccentricity from center to periphery. Wässle et al. (1989, 1990) showed that ganglion cells and cone pedicles had almost the same density at 3 mm (~15°) eccentricity in the temporal retina of *M. fascicularis*. This locus is equivalent to our present sample obtained at the same eccentricity. Boycott and Hopkins (1991) showed that some of the invaginating (ON) and flat (OFF) midget bipolar cells had synapses with two adjacent cones throughout the retina from 2.5 mm to at least 10.0 mm in macaque and vervet monkeys (*M. mulatta* and *Cercopithecus aethiops*). We occasionally observed such midget bipolar cells in our series and also found that a midget ganglion cell collects input synapses from 2 to 4 midget bipolar cells in either ON or OFF pathway (data not shown). These preliminary surveys show that the examination area is not foveal but peripheral in nature.

### 3D reconstruction using serial section transmission electron microscopy

We used a series of the electron micrograph prints acquired at 12000× (4× enlargement of 3000× negatives) for 3D reconstruction. This format provided enough open space in the sectional images to label unique letters for identification. To identify the continuity of a target process from one section to the next, we used the overlap area between adjacent cell profiles of this particular process and also all surrounding processes that were uniquely identified; we regarded the entire spatial relationship of the process with its neighbors as a specific property at each locus in every section. This method compensated for a disadvantage of our use of serial section transmission electron microscopy: successive sections could not be as completely physically registered as in serial block-face scanning electron microscopy (Helmstaedter et al., 2013). Human observers easily identified the correspondence between two characteristic patterns composed of many process profiles; some processes changed, but others remained the same. Observers also employed additional cytological clues to identify correspondence.

For automated 3D reconstruction (Mishchenko, 2009), the overlap area between adjacent cell profiles of the target process is thought to be critically important. The overlap area is largest when the process runs at a right angle to the section plane, but it becomes smaller and finally disappears as the encompassing angle approaches 0°, and thus this clue is completely absent in some longitudinally sectioned processes. To avoid this difficulty as much as possible, thinner sections (such as 20–30 nm) are required in Mishchenko's automated reconstruction approach.

However, when we expanded the observation area around the target process, because the sectional area of the target process became wider, clues about the relationship of the target process with its neighbors became rich enough to compensate for the loss in overlap area. In addition, human experts were able to evaluate the multitude of gray intensities caused by densely stained membranes in adequately thick sections. Many clues, such as the beginning or end of the process and the heading of the process, were contained in gradual changes in the finely graded images in specific places and directions. We traced every neuronal process while marking synapses and other features with colored pens on transparent sheets, drew the cell contours, and saved digitized contour lines on a personal computer using the Intuos-4 digitizer (Wacom, Saitama, Japan) and TRI/3D-SRF-R graphics software (Ratoc Systems International, Tokyo, Japan). Even after review of the graphics, precise areas of interest could be re-photographed whenever necessary by imaging the preserved sections with an electron microscope at different magnifications and tilting angles. Although the above procedure required a long time to obtain sufficient numbers of reconstructed neurons, it enabled us to determine all synaptic contacts of a given neuron as precisely as possible. Here, we present 3–5 reconstructed cells for each cell type. Furthermore, we compare our electron microscopy-based analysis with previous light microscopy-based studies of large numbers of cells at different retinal eccentricities (e.g., Wässle et al., 2009) to complement each other.

### Cluster analysis with morphological data

We reconstructed a total of 20 OFF bipolar cells from the macaque retina and 17 from the mouse retina, which included 3–5 cells of each type. A serial number was assigned to each cell that included the cell type (for example, cell DB3b-2 is the second diffuse bipolar type 3b cell included in the analysis). We acquired a dataset of four morphological variables from cells in the inner plexiform layer: (1) the level of stratification of the bipolar cell axon terminal, measured as the distance from the most distal axon terminal to the GCL, (2) the stratification thickness of the axon arbor, measured as the distance from the branching point of the axon cylinder to the axon terminal, (3) the axon arbor area projected on the horizontal plane, and (4) the number of axon synaptic ribbons. The mean and standard deviation for every variable obtained from each OFF bipolar type group are listed in Table 1. The series for mouse T1-3 and T3a-3 cells lacked a part of the axon arboreal processes (they extended in the horizontal direction beyond the series range), and thus these cells are omitted from the data in columns 3 and 4 of Table 1. The values for variables (1) and (2) from these cells are included in Table 1 because we consider them to be accurate.

**Table 1:** **Characterization of OFF bipolar cell types according to morphological characteristics of their axon terminals**.

**Bipolar cell type**	**Distance between axon and the GCL (μm)**	**Axon arbor thickness (μm)**	**Axon arbor area (μm^2^)**	**Number of ribbons**
**MACAQUE**				
DB1	23.6 ± 2.0 (4)	9.9 ± 2.6 (4)	389.8 ± 84.1 (4)	81 ± 8.2 (4)
FMB	19.1 ± 3.3 (5)	12.0 ± 3.4 (5)	70.4 ± 12 (5)	92.8 ± 7.8 (5)
DB3a	19.5 ± 0.5 (3)	11.2 ± 2.3 (3)	508.3 ± 75.2 (3)	76.7 ± 8.5 (3)
DB3b	18.6 ± 0.8 (4)	7.0 ± 0.8 (4)	235.8 ± 23.5 (4)	76.0 ± 4.2 (4)
DB2	17.1 ± 0.9 (4)	9.3 ± 1.6 (4)	154.2 ± 23.1 (4)	132.8 ± 23.2 (4)
**MOUSE**				
T1	28.8 ± 1.2 (3)	7.9 ± 0.8 (3)	222.5 ± 19.6 (2)	125.5 ± 4.9 (2)
T2	27.5 ± 0.6 (3)	11.4 ± 0.7 (3)	221.3 ± 81.8 (3)	168.3 ± 23.6 (3)
T3a	24.0 ± 1.2 (3)	9.3 ± 3.5 (3)	236.2 ± 41.9 (2)	82.5 ± 14.8 (2)
T3b	22.8 ± 0.6 (4)	10.4 ± 0.6 (4)	103.1 ± 22.2 (4)	75.8 ± 7.4 (4)
T4	21.1 ± 0.5 (4)	15.5 ± 3.1 (4)	134.3 ± 22.3 (4)	85.0 ± 9.9 (4)

*Data are expressed as mean ± standard deviation (number of cells for each type)*.

Cluster analyses (Ward's joining method) using Statistica 06J (Statsoft Japan, Tokyo, Japan) were applied to 18 macaque cells and 15 mouse cells. Macaque cell DB1-2 extended a single tiny process from the axon terminal toward the GCL, and cell FMB-2 had an axon terminal with no descending projections due to spatial hindrance by the surrounding cells. Data from these two cells severely skewed the distributions of variables (1) and (2), and so these data were omitted from the cluster analysis for macaque. Likewise, cells T1-3 and T3a-3 were omitted from the cluster analysis for mouse. We also used Statistica 06J for the Pearson's correlation test where differences for which *P* < 0.05 were considered significant.

## Results

### Classification of OFF bipolar cells

#### Nomenclature and quantitative assessment

**Figure 2:**
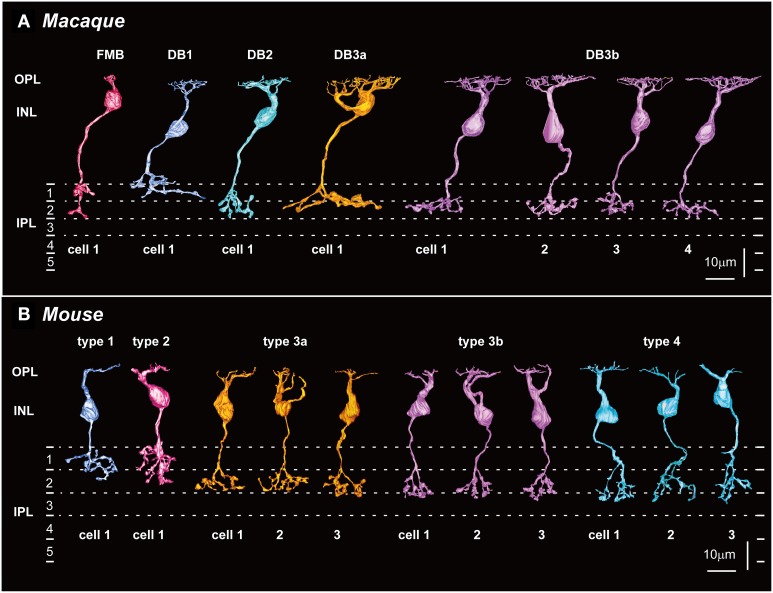
**Five types of OFF bipolar cells in macaque (A) and mouse (B) retinas**. **(A)** FMB, DB1, DB2, and DB3a cells (one example of each; left) and DB3b cells (four examples, DB3b-1 to DB3b-4) that make contact with both rods and cones in the macaque retina (right). **(B)** T1 and T2 cells (one example of each type; left), and T3a, T3b, and T4 cells (three examples of each) that make contact with both rods and cones in the mouse retina (right). Potential homologous pairs between macaque and mouse are depicted using the same colors (such as FMB and T2 in red and DB2 and T4 in light blue). OPL, outer plexiform layer; INL, inner plexiform layer; IPL, inner plexiform layer. Dotted lines indicate 0, 20, 40, and 60% depths in IPL.

Side views of OFF (cone) bipolar cells are shown in Figure 2A for macaque and in Figure 2B for mouse. The nomenclature for the macaque OFF bipolar cell types (FMB, DB1, DB2, DB3a, and DB3b) follows that previously established by Boycott and Wässle (1991), except that DB3 is further divided into DB3a and DB3b. Classification of mouse OFF bipolar cells was established by Wässle et al. (2009) as types 1, 2, 3a, 3b, and 4 (T1, T2, T3a, T3b, and T4, respectively).

First, we addressed how the previous classification based on light microscopy relates to our classification according to serial section transmission electron microscopy. For bipolar cells in the mammalian retina, side- and top-view profiles provided primary clues. The stratification level of the bipolar cell axon terminal (side view), the stratification thickness of the axon arbor (side view), and the axon arbor area (top view) in the inner plexiform layer have been measured in many studies to distinguish bipolar cell types (e.g., Kolb et al., 1981; Cohen and Sterling, 1990; Boycott and Wässle, 1991; Euler and Wässle, 1995; Badea and Nathans, 2004; Ghosh et al., 2004; Li et al., 2004; Pignatelli and Strettoi, 2004). For comparison with previous studies, we adopted these three structural variables and further included the total number of axon synaptic ribbons in our analysis. Many other ultrastructural features of bipolar cells, such as the distribution of dendritic contacts with cones (Hopkins and Boycott, 1997) and synaptic contacts with amacrine and ganglion cells, are beyond the scope of this study, but will be presented in future reports.

#### Axon stratification level and thickness

**Figure 3:**
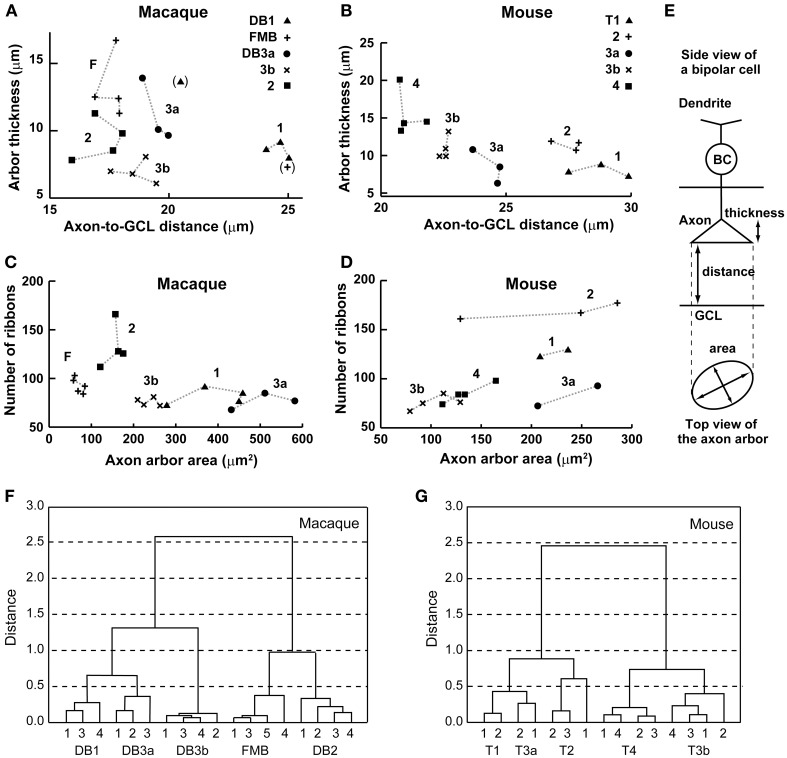
**Scatter plots and cluster analyses**. **(A–D)** Scatter plots of axon-to-GCL distance vs. axon arbor thickness (**A**, macaque, 20 cells; **B**, mouse, 17 cells) and axon arbor area vs. the number of axon ribbons (**C**, macaque, 20 cells; **D**, mouse, 15 cells). This dataset also appears in Table 1. **(E)** Explanatory illustrations for three morphological variables. BC, bipolar cell. **(F,G)** Cluster analyses of bipolar cell types in four-dimensional space (distance, thickness, area, and number of ribbons) using Ward's joining method (ordinate, Euclidian distance; abscissa, serial number of each cell). The macaque dataset consists of 18 cells (cells DB1-2 and FMB-2 are omitted due to their exceptional values; parentheses in **A**). The mouse dataset consists of 15 cells (cell T4-2 is included but cells T1-3 and T3a-3 are omitted).

From the side-view images, we acquired the axon-to-GCL distance and the axon arbor thickness (Table 1; Figures 3A,B). In the macaque retina, the stratification level of axon terminals did not follow their numerical order (Figure 2A; Table 1). The axon terminals of DB1 cells were located at the highest level (longest distance from the axon terminal to the GCL), but those of DB2 cells were located at the lowest level (shortest distance from the axon terminal to the GCL). The level of DB3a terminals was slightly higher than that of DB3b terminals, both of which were close to that of FMB terminals (Table 1; Figure 3A). FMB cells usually extended a process from the axon terminal toward the GCL to contact the entire span of the midget ganglion cell dendrites; consequently, the terminal stratification level of these cells was deep (Figure 2A).

In the mouse retina, Ghosh et al. (2004) primarily characterized mouse bipolar cell types based on axon terminal stratification levels. Accordingly, the nominal order from T1 to T4 corresponded in descending order with the terminal inner plexiform layer level measured by the axon-to-GCL distance (Figure 2B). Thus, stratification level was the most effective classification variable among the four variables assessed here (Table 1). Nevertheless, the difference in the distance from the axon terminal to the GCL seemed to be insufficient to distinguish T1 from T2 cells or T3a from T3b cells because the mean differences were too small compared to their standard deviations (Table 1; Figure 3B). Therefore, other variables were needed for more precise discrimination.

Axon arbor thickness was defined as the distance from the first axon branch point on side view to the distal axon terminal (Table 1). In the macaque retina, this variable distinguished DB3a from DB3b cells because the axonal arbor was considerably thicker in DB3a cells, but no other pairs of OFF bipolar cell types were distinguished. In the mouse retina, T1 cells had the thinnest axon arbor and T4 the thickest axon arbor; thus, this variable distinguished T1 from T2, T3b, and T4 cells as well as T4 from all other types of OFF bipolar cells (Figures 3A,B).

#### Axon arbor area and number of ribbon synapses

**Figure 4:**
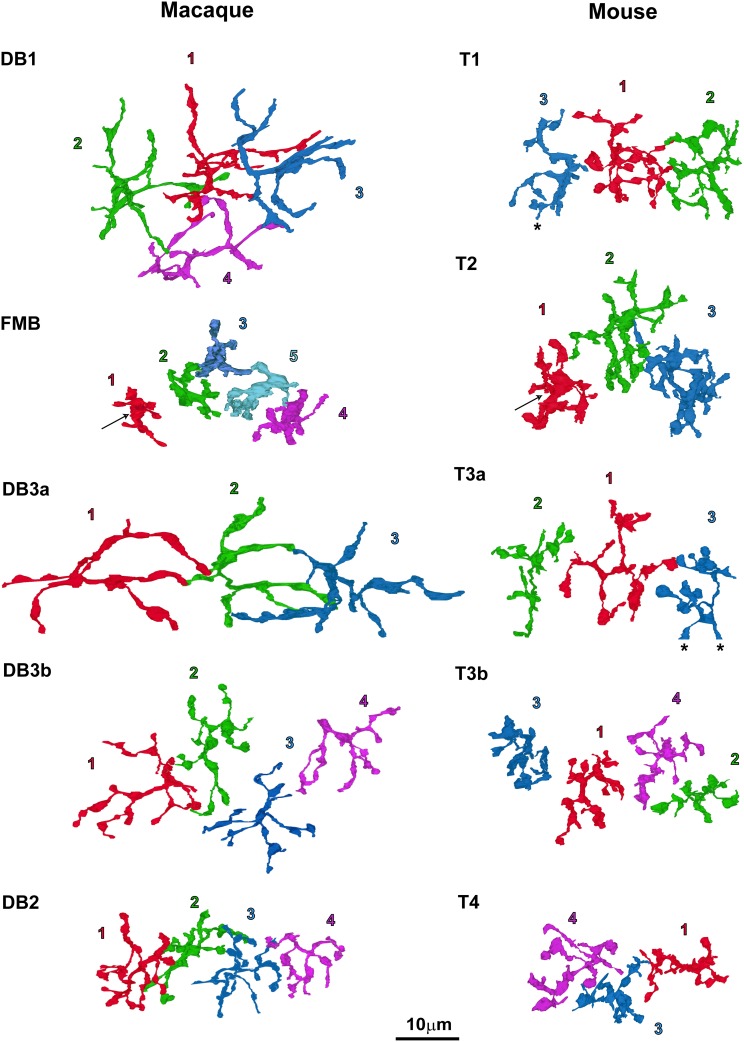
**Top-view profiles of the axon terminal arbors of the five types of OFF bipolar cells in the macaque retina (left; 20 cells in total) and the mouse retina (right; 16 cells in total)**. For each cell type, 3–5 neighboring cells of the same type are displayed with the serial numbers used in other figures. T4-2 is omitted from this figure because it was located away from the other three cells of this type. The arrows at FMB and T2 denote a central wide area of the arbor silhouette. T1-3 and T3a-3 denotes the end of the series. Cells are colored for clarity.

Top views of the OFF bipolar cell axon terminals in macaque and mouse are displayed in Figure 4. We calculated the axon arbor area (Table 1) from these images, assuming that the outer circumference is an ellipse (Figure 3E). As an additional ultrastructural variable, we counted the number of axon synaptic ribbons (Table 1) within the axon arbor area. These two variables are displayed for macaque (Figure 3C) and mouse (Figure 3D).

Axon arbor area was the most effective variable for classifying macaque OFF bipolar cell types (Figure 3C). Axon arbor area increased in the order of FMB, DB2, DB3b, DB1, and DB3a. For example, DB3a cells had a mean arbor area twice that of the DB3b type (508 vs. 236 μm^2^), and DB3b cells typically had considerably larger arbor areas than DB2 cells (154 μm^2^). FMB OFF bipolar cells had the smallest mean arbor area (70 μm^2^), allowing easy discrimination from all other types. Thus, with the exception of types DB1 and DB3a, quantitative differences exist in the axon arbor areas of all other types. Although the difference in the axon arbor areas of DB1 and DB3a cells was small, the shapes of individual axon arbors and the patterns of multiple axon arbors of the same type enabled us to distinguish between types DB1 and DB3a (Figure 4, left).

Ghosh et al. (2004) classified T3 as a single mouse OFF bipolar cell type, but also suggested the possibility of further divisions based on morphological variability within this group. Mataruga et al. (2007) divided T3 into two types with antibodies against HCN4 for T3a and PKARIIβ for T3b. Using the same immunostaining method, Wässle et al. (2009) showed that the arbor area encompassing axon terminals is wider in T3a cells than in T3b cells. We also utilized this difference in axon arbor area as a classification criterion for splitting T3 into T3a cells (mean axon arbor area 236 μm^2^) and T3b cells (mean axon arbor area 103 μm^2^).

The number of ribbons ranged from 70 to 100 for all types of macaque OFF bipolar cells except DB2, which had ~130 ribbons. The numbers of ribbons varied to a much greater extent in the mouse OFF bipolar cells, from 70 to 90 for T3a, T3b, and T4, 130 for T1, and 170 for T2. The number of ribbons correlated with the axon arbor area (Pearson's correlation coefficient: *r* = 0.63, P^*^ = 0.011, *n* = 15) in the mouse retina (Figure 3D) but not (*r* = −0.41, *P* = 0.063, *n* = 20) in the macaque retina (Figure 3C). The number of ribbons yielded only moderate discriminative power, distinguishing 6/10 pairs for both macaque and mouse (Table 1; Figures 3C,D).

#### Cluster analysis for distinguishing OFF bipolar cells

No single morphological variable distinguished all OFF bipolar cell types in either species. For example, macaque DB3a cells had longer axon-to-GCL distances than DB2 cells, but these distances were too similar to those of DB3b cells to distinguish between cell types. However, DB3a cells had axon arbor areas that were twice as wide as those of DB3b cells (Figures 3A,C). Mouse T1 and T2 cells were not distinguishable by the distance from the axon terminal to the GCL, but axon arbor thickness and number of axon ribbons allowed their classification (Figures 3B,D).

In contrast to single variables, cluster analysis of all four variables (normalized to the highest within-class value) in multidimensional space yielded five distinct OFF bipolar cell types in both species (Figures 3F,G). DB1 and DB3a were close to each other in this analysis mainly because these types had the second-largest and largest axon arbor areas, respectively, and the second-smallest and smallest ribbon numbers, respectively. FMB and DB2 closely clustered because they had the smallest and second-smallest axon arbor areas, respectively, and the second-largest and largest ribbon numbers, respectively (Table 1). Likewise, T1 and T3a closely clustered mainly because they had the smallest and second-smallest axon arbor thicknesses, respectively, and the second-largest and largest axon arbor areas, respectively. T3b and T4 clustered closely because they had the smallest and second-smallest axon arbor areas, respectively, and the second-shortest and shortest axon-to-GCL distances, respectively (Table 1). These clustering results are substantiated by top-view profiles in which the neighboring axon arbors of the same type were tiled in this arbitrarily chosen patch of retina (Figure 4). This tiling according to cell type is expected to occur when all OFF bipolar cell types are properly classified.

We noticed an interesting inverse relationship between axon-arbor thickness and axon-arbor area (Table 1; Figures 3A–D). In the mouse retina, T1 cells had the smallest thickness but the second-largest area; likewise, T3a cells had the second-smallest thickness but the largest area. In contrast, T3b cells had the third largest thickness but the smallest area, and T4 cells were the thickest, but had the second-smallest area. Roughly speaking, T1 and T3a cells are characterized by axon arbors with small thickness and large area, but T3b and T4 cells are characterized by axon arbors with large thickness and small area. T2 cells do not fit into this scheme. In the macaque retina, FMB cells had the largest thickness but the smallest area. Thus, FMB is the type best characterized by the inverse relationship between large thickness and small area. However, this inverse relationship was not detected in the axon arbors of the four other macaque cell types.

### Electron micrographs of basal synaptic contacts and gap junctions

#### Synapses at rod and cone terminals

**Figure 5:**
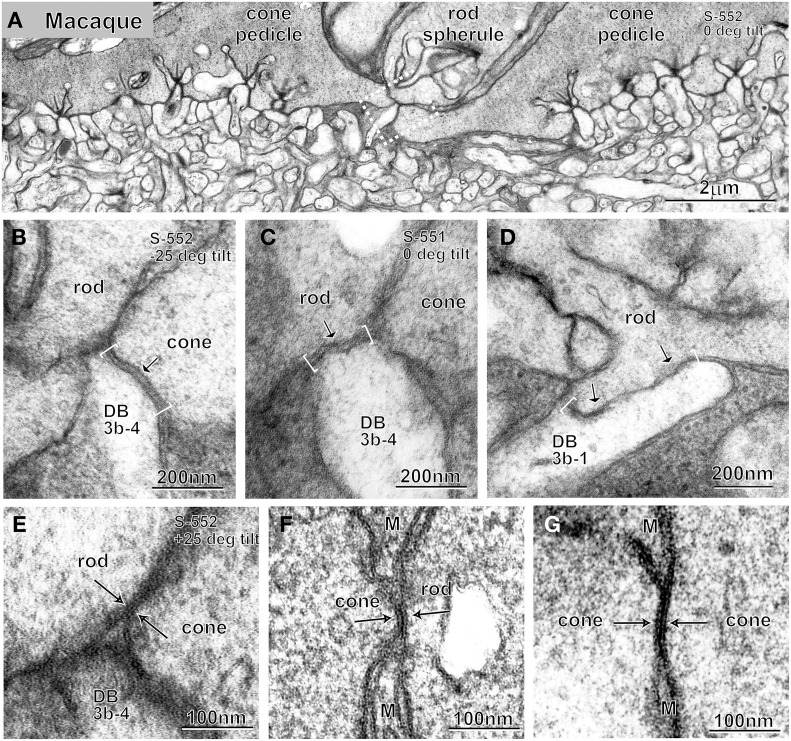
**Electron micrographs at low (A) and high (B–G) magnifications**. A rod spherule flanked by two cone pedicles **(A)**, basal contacts of DB3b cells with cone **(B)** and rod **(C,D)** terminals, and rod-cone **(E,F)** and cone-cone **(G)** gap junctions. Pre- and post-synaptic membranes are smooth and arched with a cleft of ~20 nm at cone basal contacts (bracket and arrow; **B**) but ragged at rod basal contacts (brackets and arrows; **C,D**). Gap junctions display a characteristic structure of three (thin, thick, and thin) dark lines (arrows; **E–G**). Micrographs **(B,C)** are adjacent sections of the same locus; micrographs **(B,E)** are a single section (S-552) of the same locus (dotted rectangle in **A**) displayed at different angles (−25° and +25°). M, Müller cell process.

Dendrites of DB3b cells made contact with both rods and cones at the photoreceptor basal surface in the macaque retina. For instance, cell DB3b-4 extended a dendritic tip at a meeting place of two pedicles and one rod (Figure 5A) and made contact with a cone (Figure 5B) at a concave indentation of the presynaptic membrane. At this contact point, both pre- and post-synaptic membranes were electron dense, and the cleft was of constant width (~20 nm). In the adjacent retinal section containing the dendritic tip (Figure 5C), the DB3b-4 cell made contact with the basal surface of a rod. At this synapse, the presynaptic membrane was not concave, the pre- and post-synaptic membranes were not smooth, and the cleft varied in width. At another synapse involving cell DB3b-1 and a rod, two additional features were evident (Figure 5D): the contact length greatly varied (this contact was much wider than others) and regions of high membrane electron density were intermittent (arrows in Figure 5D). These features were previously noted in rod basal contacts (Hack et al., 1999; Tsukamoto et al., 2001). In the proximity of the DB3b-4 dendritic tip (Figure 5E), we observed a rod-cone gap junction by tilting the section 552 by 50° relative to the orientation in Figure 5B. Rod-cone gap junctions were present most often between the rod basal surface close to its invagination aperture and the fine process emanating from the cone terminal (Tsukamoto et al., 2001), but they were also observed between terminals of neighboring rods and cones (Figure 5F). In fenestrations lacking the flattened processes of Müller glia, the membranes of rod spherules and cone pedicles made contact with each other, forming a structure with three (thin, thick, and thin) parallel electron-dense lines, which is the ultrastructural hallmark of gap junctions. Cone-cone gap junctions were also observed between every pair of adjacent cone pedicles (Figure 5G).

#### Localization of rod-rod gap junctions

**Figure 6:**
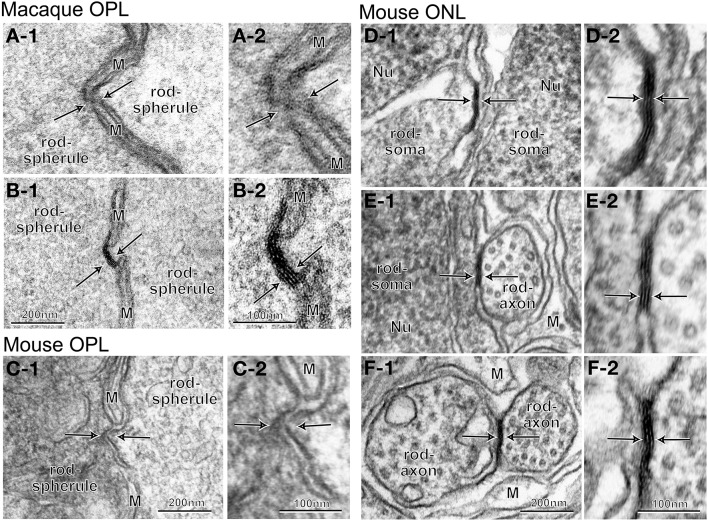
**Electron micrographs of the interface between adjacent rod cells at different levels**. **(A–C)** No characteristic structures indicative of gap junctions (arrows) are seen between rod spherules. Pinpoint contacts of two rod membranes occur where thin, intervening processes of Müller cells are blurry **(A)**, continuous (**B**, five lines), or discontinuous **(C)**. **(D–F)** Typical gap junctions (arrows) are found between rod somas **(D)**, somas and axons **(E)**, or axons **(F)**. M, Müller cell process.

We examined whether gap junctions exist between adjacent rod terminals in the OPL in both macaque and mouse retinas by using their radial sections in series. In the macaque retina, we surveyed all 65 contiguously distributed rod terminals within an arbitrarily chosen area. We found 57 sites where the opposing membranes of two adjacent rod terminals appeared to be darker and closer than other neighboring structures (Figures 6A-1,B-1). Each rod had 1–5 such sites (mean ± standard deviation, 1.8 ± 0.8). These candidate gap junction sites were inspected at a higher magnification with section tilting to observe the length of the membrane surface in cross section. One typical case was a blurred image at the intersection of rod and glial membranes (Figure 6A-2). The other typical case was a pattern of five parallel dense lines (Figure 6B-2) that appeared to consist of four membranes (two outer membranes from rods and two inner membranes from glial cells) in intimate contact. Neither case exhibited the pattern of three parallel dense lines that are characteristic of gap junctions (Figure 5F). Likewise, in the mouse retina, we surveyed 60 contiguously distributed rod terminals within an arbitrarily chosen area. We found 50 sites with the opposing membranes of two adjacent rod terminals, 1–4 such sites per rod (1.5 ± 0.9). Those candidate gap junction sites (Figure 6C-1) were inspected at a higher magnification with section tilting. One typical case showed two rod cell membranes, one convex but the other concave, that were slightly separated from each other at the fenestration of Müller cell processes (Figure 6C-2). In this survey, we located no definite rod-rod gap junctions.

Next, we examined the existence of rod-rod gap junctions in the ONL by analyzing a series of tangential sections (containing the ONL) of the retina from a male C57BL/6J mouse. Slender axons from rod and cone cells passed through the interstitial spaces among multiple tiers of densely packed rod and cone cell somas toward the OPL. Those photoreceptor cell elements were ensheathed by the flattened processes of Müller cells, which were sparser than those in the OPL. We readily identified gap junctions between adjacent rod somas (Figure 6D), a rod soma and an axon (Figure 6E), and two rod axons (Figure 6F). These gap junctions were distributed across the ONL from the outer limiting membrane to the border between the ONL and the OPL. Taken together, these data indicate that rod-rod gap junctions are rare in the OPL in the macaque and mouse retinas but frequent in the ONL in the mouse retina.

### Distribution of rod basal contacts with the OFF bipolar dendrites

**Figure 7:**
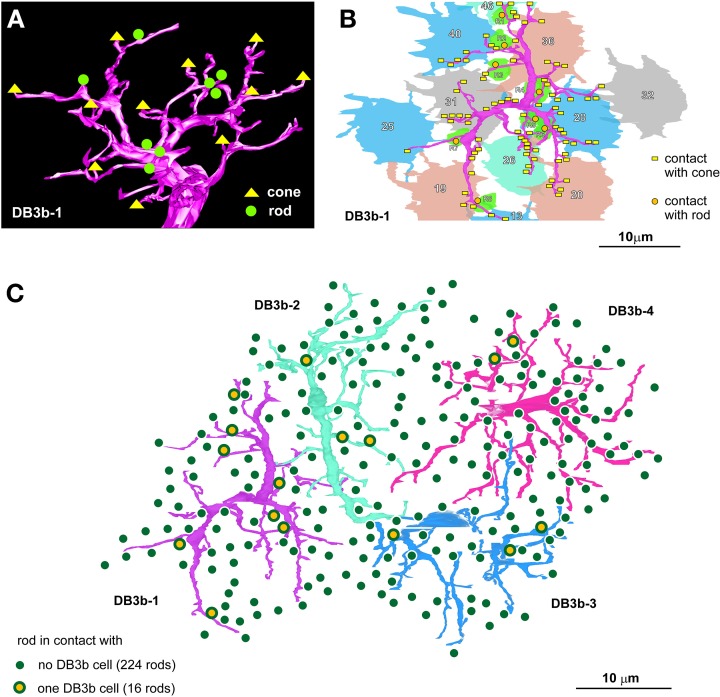
**A macaque OFF bipolar type contacts both rods and cones**. **(A)** Dendritic connections of cell DB3b-1 with 8 rods and 11 cones. **(B)** One basal contact is formed with each rod, but different numbers of contacts are formed with each cone. **(C)** Distribution of 240 rods covered by the dendritic fields of four DB3b cells (cells are colored for clarity). The percentage of the rods that are in contact with any of these DB3b cells is 6.7%.

The DB3b type of OFF bipolar cells formed connections with both rods and cones in the macaque retina. For example, cell DB3b-1 made contact with eight rods and 11 cones (Figure 7A). Each of DB3b cells 1-4 made contact with 4.0 ± 2.7 rods and 9.0 ± 1.4 cones. Cell DB3b-1 made contact with each rod via a single basal contact, but with each cone with 1–19 basal contacts (Figure 7B). Each DB3b cell made contact with a single cone via 9.4 ± 6.3 basal contacts (*n* = 36). It was evident that not all rods formed this type of connection with DB3b cells. To assess the proportion of rods that made contact with DB3b cells, we examined an area where the dendrites of four DB3b cells covered the area occupied by contiguously distributed rods (Figure 7C). In this area, every rod was in close proximity to at least one of the four DB3b cells. However, only 16 rods made contact with one of the four DB3b cells, whereas the remaining 224 rods did not. Thus, 6.7% of rods formed a basal contact with an OFF bipolar cell in this locus of the macaque retina.

**Figure 8:**
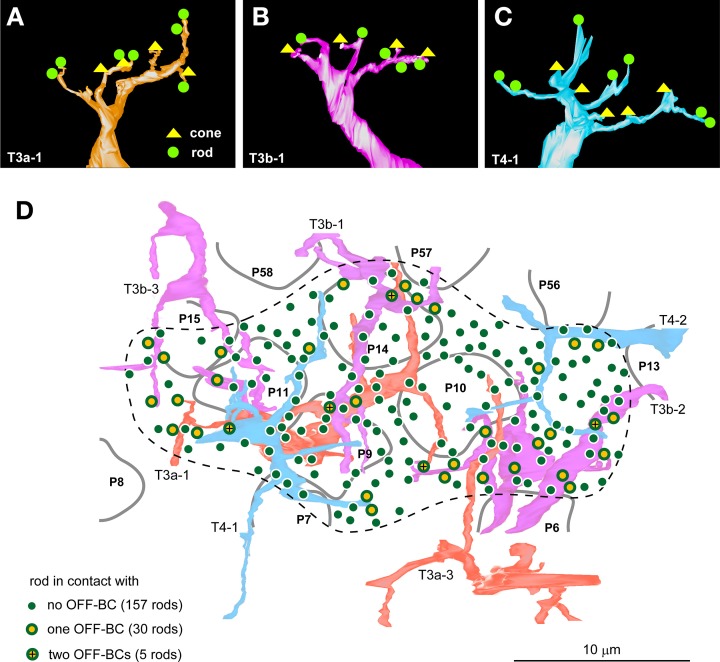
**Three mouse OFF bipolar types contact both rods and cones**. The basal contacts of rods and cones with cell T3a-1 (**A**; seven rods and four cones), cell T3b-1 (**B**; five rods and four cones), and cell T4-1 (**C**; seven rods and five cones) in the mouse retina. **(D)** Distribution of 192 rods covered by the dendritic fields (dotted contour line) of two T3a, three T3b, and two T4 cells. The percentage of the rods making contact with one or two T3a, T3b, and T4 cell types is 18.2%. Four central cone pedicles (P9, P10, P11, and P14) make contact with all three types of OFF bipolar cells.

We also examined the photoreceptor connectivity of the OFF bipolar cell dendrites in the mouse retina and found that T3a, T3b, and T4 cells formed basal contacts with both rods and cones. Every cell of the three OFF bipolar cell types that were examined (3–4 cells per type) formed such contacts with variable numbers of rods and cones. The most representative case for each OFF bipolar cell type is displayed in Figures 8A–C; however, the dendrites of 4/11 examined cells were only partially reconstructed within the series. Therefore, we generated no mean values of the numbers of connected rods and cones for each OFF bipolar cell type. Instead, we focused our analysis on a certain area in which the dendrites of T3a, T3b, and T4 cells intermingled and covered all contiguously distributed rods (Figure 8D). Inside the dotted contour of Figure 8D, four cone pedicles (P9, P10, P11, and P14) were in contact with these three types of OFF bipolar cells, indicating that rod spherules in the same area were also in close proximity to these three types of OFF bipolar cells. Thirty rods contacted one of the OFF bipolar cells, and five rods contacted two OFF bipolar cells of different types; the remaining 157 rods had no contact with any OFF bipolar cell. Thus, 15.6% of rods formed a basal contact with an OFF bipolar cell, and 2.6% of rods made contacts with two OFF bipolar cells of different types. In total, 18.2% of rods had direct connections to OFF bipolar cells.

## Discussion

### Homologous OFF bipolar cell types in the macaque and mouse retinas

In our recent preliminary report (Tsukamoto and Omi, 2013), which was conducted independently of Puthussery et al. (2013), we identified a new type of DB OFF bipolar cell in addition to the three previously described DB cell types in macaque retina, and demonstrated that this new type of OFF bipolar cell makes basal contact with both rods and cones. The shape of the axon terminals of this new type was similar to that of DB2. We previously called this new cell type DB2b, but here we have adopted DB3b as its name throughout this article. When Boycott and Wässle (1991) morphologically classified macaque bipolar cells, they reported cell densities of 2935/mm^2^ for DB2 cells and 968/mm^2^ for DB3 cells. Thus, DB2 cells were three-fold more numerous than DB3 cells, probably due to a difference in cell size and/or a mixture of multiple types. It was highly likely that the newly discovered type of DB cells had been combined with DB2 cells as a single type in Boycott and Wässle's classification. Puthussery et al. (2013) named the newly discovered type DB3b, probably because the axon terminals of these cells were located almost at the same level as those of DB3 cells. Our observation that macaque DB3a and DB3b cells are homologous to mouse T3a and T3b cells, respectively, supports the use of this nomenclature; the axon arbor areas of “3a” cells are approximately double those of “3b” cells in both species.

Previous morphological and immunohistochemical analyses, the recent discovery of the DB3b type, and our current comparative morphometric study indicate that the mouse and macaque monkey retinas possess five homologous and distinct OFF bipolar cell types. In their respective species, cell types DB1 and T1 had the highest axon stratification level and the second-largest axon arbor area, whereas types DB2 and T4 had the lowest stratification level and the second-smallest axon arbor area (Figure 2; Table 1). If we tentatively eliminate types FMB and T2 from the classification samples, a sequence of DB1, DB3a, DB3b, and DB2 for macaque and a sequence of T1, T3b, T3b, and T4 for mouse reflect axon-to-GCL distance in descending order. These orderly distances may indicate the homologous correspondence between types DB1 and T1, DB3a and T3a, DB3b and T3b, and DB2 and T4. The remaining cell types in each species, FMB in macaque and T2 in mouse, therefore may correspond to each other.

FMB cells had the smallest axon arbor area in macaque, whereas T2 cells had the third-largest axon arbor area, which was more than twice as large as the smallest axon arbor area of T3b cells in mouse. However, Ghosh et al. (2004) suggested that macaque FMB cells and mouse T2 cells are homologous, because both displayed recoverin immunoreactivity (Milam et al., 1993; Euler and Wässle, 1995; Haverkamp et al., 2003a,b). In this case, morphological classification could be complemented by immunohistochemical approaches. It is conceivable that FMB cells drastically change their morphology from the common ancestral type shared by T2 cells in order to adapt to high-acuity vision under bright daylight conditions, with no critical changes in immunochemical properties. In this context, although our side-view profile of a T2 cell (Figure 2B) may not exactly resemble that displayed by Ghosh et al. (2004), possibly due to inherent morphological variability, it is very similar to that depicted by Ivanova et al. (2006). The axon arbor profiles of T2 cells and FMB cells were similar in appearance from the top view (Figure 4); at the center of the axon arbor area of both species, a round wide area (denoted by arrows) was seen. Similarly, a skirt-like stout portion was evident at the branching point of the axon arbor in both cells in the side view (Figure 2). This particular feature was not identified in other OFF bipolar cell types, which instead exhibited a few slender processes that diverged from the branching point of the axon. All these findings are consistent with the interpretation that the mouse T2 cell is homologous to the macaque FMB cell.

### Comparison of rod-to-bipolar-cell connections in the macaque and mouse retinas

Based on the present results, we now add macaque monkeys to the list of mammals, such as mice, rats, cats, and rabbits, that harbor the third rod signaling pathway. However, we note that the pathway that connected rods to OFF bipolar cells appeared to be less distributed over the macaque retina. In mouse, three types of bipolar cells (T3a, T3b, and T4) were involved in this pathway, whereas in macaque, only one type of bipolar cell (DB3b) made contact with rods. In accordance with this difference, ~18% of mouse rods formed contacts with OFF bipolar cells, compared with only 7% of macaque rods.

In the mouse retina, every T7 ON bipolar cell that we previously examined (Tsukamoto et al., 2007) made direct synaptic contacts with at least two rods, and on average 1–2% of the rods had direct contacts with T7 ON bipolar cells. In contrast, in the present investigation we identified no contacts between the dendrites of one ON bipolar cell of each type and rods in the macaque retina (data not shown). Therefore, it is likely that the proportion of rods that directly contact ON bipolar cells is exceptional, if such direct contacts exist. These two species-specific differences suggest that, compared to the macaque retina, the mouse retina is better adapted to nocturnal conditions through its implementation of more channels for rod signals.

Tsukamoto et al. (2001) showed that the rods in the mouse retina that contacted OFF bipolar cells were coupled with several neighboring rods by gap junctions, possibly to integrate rod signals. In the macaque retina, Hornstein et al. (2005) reported rod-rod tracer couplings with Neurobiotin, as well as rod-rod electrical couplings; those gap junctions were located in regions containing rod somas and axons. We observed that gap junctions between rod spherules were rare in the OPL of the macaque retina, confirming the findings of Raviola and Gilula (1973). Our further investigation into both the OPL and the ONL of the mouse retina revealed that rod-rod gap junctions were rare in the OPL but occurred frequently between adjacent somas, somas and axons, and adjacent axons, which were distributed from the outer limiting membrane to the ONL-OPL border, confirming and extending the previous findings (Tsukamoto et al., 2001). All the above findings strongly suggest that rod-rod gap junctions are localized in the ONL in both macaque and mouse retinas. Rod-rod gap junctions participate in all three rod signaling pathways as a kind of preprocessing of the output from rod (chemical and electrical) synaptic terminals. Where the third rod signaling pathway is concerned, a small portion (~18% for mouse, ~7% for macaque) of rods may electrically couple with other surrounding rods before directly contacting OFF bipolar cells. Despite the low percentage of rods that directly contact OFF bipolar cells, transmitted signal may be effectively driven by a much larger portion of the rods under certain mesopic conditions. Therefore, we cannot rule out the possibility that the third rod signaling pathway is a necessary device in an overall, intricate architecture that remains to be further elucidated.

### Physiological significance of the direct pathway from rods to OFF bipolar cells

Three known rod pathways are thought to differ in temporal signaling characteristics as well as in light sensitivity. Response speed is constrained by the number of synaptic transmission steps between the photoreceptor and bipolar cells. Ignoring rod-rod coupling, the first pathway consists of three steps: two chemical synapses from rod to rod and bipolar cells to AII amacrine cells, and a third chemical synapse from AII cells to OFF cone bipolar cells (or one parallel electrical synapse from AII cells to ON cone bipolar cells). The second pathway consists of two steps: one electrical synapse from rod to cone and one chemical synapse from cone to OFF (or in parallel ON) cone bipolar cells. The third pathway consists of only one step, a chemical synapse from rod to OFF cone bipolar cells. Soucy et al. (1998) first suggested the existence of direct synaptic contacts between rods and OFF cone bipolar cells in order to explain the fast response of ganglion cells to OFF stimuli in the coneless mouse. In human psychophysical and electroretinographic studies, a loss of flicker visibility and electroretinographic amplitude at frequencies near 15 Hz were shown to result from cancelation between sensitive “slow” and insensitive “fast” rod signals, with a time difference of ~33 ms (Stockman et al., 1995; Sharpe and Stockman, 1999). The sensitive “slow” rod signals could be unequivocally ascribed to the first pathway. The insensitive “fast” rod signals were thought to occur through the second pathway (via rod-cone gap junctions), according to the knowledge at that time. However, there remained a puzzling phenomenon: this “fast” signal was present even in an achromatic observer that lacked functioning cones (Stockman et al., 1991). It seems unlikely that a full range of the “first” signal can be solely ascribed to the low percentage of rods contacting DB3b cells in the third pathway. Nevertheless, these psychophysical and electroretinographic phenomena should be reconsidered in a more comprehensive framework that includes all three pathways.

Electrophysiological experiments have demonstrated that the third pathway is functional, with sensitivity and dynamics that are distinct from the other two pathways in the mouse retina (Field and Rieke, 2002; Pang et al., 2010). This pathway is thought to exhibit a light sensitivity that is intermediate between those of the first and the second pathways. The first and most sensitive pathway may work alone at the low scotopic level, whereas the second and third pathways may be activated in sequence as the light intensity increases. However, these multiple pathways appear to operate in parallel, according to a recent study by Ke et al. (2014); in the mouse retina, under a high mesopic level in which the second pathway (via rod-cone gap junctions) is fully activated, the role of the first (rod bipolar cell to AII amacrine cell) changed from encoding the absorption of single photons to encoding contrast modulations. Under the incessantly changing environmental light conditions at the scotopic and mesopic levels, the third pathway may also work simultaneously with the first and second pathways, but with different dynamics. This circuitry of multiple channels with different temporal characteristics for rod signals exemplifies the functional significance of parallel processing.

## Author contributions

Yoshihiko Tsukamoto designed this study, took micrographs, acquired data, and wrote the manuscript. Naoko Omi took micrographs, acquired data, and checked the manuscript.

### Conflict of interest statement

The authors declare that the research was conducted in the absence of any commercial or financial relationships that could be construed as a potential conflict of interest.
